# Attitudes and Perceptions Toward Hand Hygiene Among Nursing Students and Nurses: A Cross‐Sectional Comparative Survey

**DOI:** 10.1111/jan.17076

**Published:** 2025-05-28

**Authors:** Per‐Ola Blomgren, Lisa Hultin, Johan Westerbergh, Katarina Hjelm

**Affiliations:** ^1^ Department of Public Health and Caring Sciences Uppsala University Hospital Uppsala Sweden; ^2^ Uppsala Clinical Research Center, Uppsala University Uppsala Sweden

**Keywords:** attitudes, comparative study, cross‐sectional survey, hand hygiene, infection prevention, nursing, nursing students, registered nurses

## Abstract

**Aim:**

To describe and compare attitudes toward hand hygiene and the perceived effectiveness of prevention methods among nursing students and registered nurses at a university and its affiliated university hospital.

**Design:**

A descriptive cross‐sectional comparative survey.

**Methods:**

A total of 201 first‐ and final‐semester nursing students and registered nurses completed the World Health Organisation's ‘Perceptions Survey for Health‐Care Workers’. The survey examined perceptions on hand hygiene, patient safety and the usefulness of improvement measures. Responses were analysed using descriptive statistics.

**Results:**

Nursing students consistently rated the importance of hand hygiene and related interventions higher than registered nurses. Students particularly emphasised the availability of hand disinfectants, ongoing education and supportive leadership. Both groups acknowledged the role of management support, regular feedback and organisational policies in reinforcing optimal hand hygiene.

**Conclusion:**

Differences in attitudes between nursing students and registered nurses underscore the need for ongoing education, strong managerial involvement and supportive policies to sustain adherence. Strengthening these factors can help maintain positive perceptions formed during training and enhance patient safety in clinical practice.

**Implications for the Profession and/or Patient Care:**

Educational curricula and workplace strategies that prioritise hand hygiene may help lower healthcare‐associated infections. Management‐led feedback, continuous training and accessible hand hygiene resources offer additional support for safe patient care.

**Impact:**

*What problem did the study address?* Low adherence to hand hygiene is a key driver of preventable infections. *What were the main findings?* Nursing students rated hand hygiene and improvement measures more highly than registered nurses, highlighting a need for strategies that sustain positive attitudes during the transition from education to clinical practice. *Who will benefit?* Nurse educators, clinical leaders and healthcare workers can use these findings to improve infection prevention across educational and practice settings.

**Reporting Method:**

We adhered to STROBE guidelines for cross‐sectional research.

**Patient or Public Contribution:**

No patients or members of the public were involved in designing or conducting this study, which focused on perceptions of nursing students and registered nurses.

**Summary**

- What does this paper contribute to the wider global clinical community?
  ○ Provides comparative insights into how hand hygiene attitudes differ between novices and experienced nurses.
  ○ Highlights the value of ongoing managerial support and education in reinforcing best practices.
  ○ Encourages bridging the gap between theoretical knowledge and clinical realities to enhance patient safety.

## 1 Introduction

Healthcare‐associated infections (HAIs) pose a significant threat in healthcare settings, affecting patients, visitors and healthcare workers (HCWs) alike (OECD 2020). In combating these infections, hand hygiene (HH) emerges as a simple yet effective strategy, with nurses playing a pivotal role in ensuring HH compliance and reducing HAIs (World Health Organization and WHO Patient Safety 2009; ICN—International Council of Nurses 2023). Despite established guidelines, HH adherence remains suboptimal, influenced by behavioural, organisational and educational factors (World Health Organization and WHO Patient Safety 2009; World Health Organization 2009; Erasmus et al. 2010; Pittet et al. 2000).

Previous research highlights that HCWs' perceptions and attitudes strongly influence HH practices (Erasmus et al. 2010; Pittet et al. 2000; Afework and Tamene 2025). Nursing students and registered nurses (RNs) represent two groups at different stages of professional development; students are still forming their infection prevention behaviours, while RNs have established routines shaped by clinical experience. No previous studies directly comparing HH perceptions between nursing students at different points in their education and practicing RNs were identified in the literature review. However, studies have examined HH perceptions within various HCW groups, including nursing students (Zimmerman et al. 2020; Barrett and Randle 2008; Labrague et al. 2018; Linnik et al. 2024), RNs, physicians and other clinical staff (Afework and Tamene 2025; Tan and Olivo 2015; Sopjani 2016; Dehghan Manshadi et al. 2022; Oh 2019), revealing differences in adherence and attitudes. These findings suggest that clinical experience and context may shape HH perceptions and attitudes. Yet, the specific comparison between nursing students at different stages of education and practicing RNs remains unexplored, leaving a gap this study aims to address.

Understanding these differences is essential for improving HH training and adherence strategies in both education and clinical practice. This study addresses this gap by examining HH attitudes among nursing students and RNs, using the World Health Organisations (WHO) ‘Perceptions Survey for Health‐Care Workers’ (WHO 2009a). Previous findings have demonstrated that knowledge levels differ between nursing students and RNs (Blomgren et al. 2024), raising the question of whether differences also exist in their perceptions and attitudes toward HH as these are based on their knowledge (Hadziabdic et al. 2020). This study focuses on studying these potential differences, hypothesizing that nursing students and RNs differ in how they perceive preventive measures and HH attitudes.

## 2 Background

HAIs remain a persistent challenge in healthcare, with HH recognised as the most effective measure to prevent transmission (World Health Organization and WHO Patient Safety 2009). To improve HH adherence, the WHO developed the Multimodal Hand Hygiene Improvement Strategy, which targets behaviour change through five core components: system change, training, monitoring, reminders and safety culture (World Health Organization 2009). Research demonstrates that interventions addressing multiple behavioural determinants are more effective than singular approaches (Luangasanatip et al. 2015; Huis et al. 2012).

The Theory of Planned Behaviour (TPB) provides a useful framework for understanding HH adherence. According to TPB, three primary factors shape behaviour: attitudes, subjective norms and perceived behavioural control (Ajzen 1991). In the HH context, attitudes refer to HCWs' beliefs about the effectiveness and necessity of HH, subjective norms reflect social and professional expectations and perceived behavioural control relates to the ability to perform HH given external constraints. Studies have shown that stronger perceived control and positive attitudes are associated with higher HH adherence (Sax et al. 2007; Braithwaite et al. 2017). In this study, the TPB (Ajzen 1991) was used as a theoretical lens to relate HH perceptions among nursing students and RNs to the constructs of attitudes, subjective norms and perceived behavioural control.

Beliefs, which shape attitudes, develop through socialisation with peers, colleagues and institutions such as healthcare organisations and universities (Ajzen 1991). However, the foundation of these beliefs lies in an individual's knowledge of the subject (Hadziabdic et al. 2020). Addressing knowledge gaps can therefore influence perceptions and attitudes toward previously mismanaged behaviours, including HH. Our earlier study showed that knowledge disparities exist between nursing students and RNs, with first‐semester students demonstrating significantly lower knowledge (Blomgren et al. 2024) than RNs, which could affect their beliefs and therefore attitudes.

Further complicating HH adherence is the variation in knowledge and perceptions across different HCW groups. While formal education provides a theoretical foundation for infection prevention, real‐world practice shapes HH attitudes over time (Zimmerman et al. 2020; Barrett and Randle 2008). Nursing students, who are still in training, may develop different perspectives on HH compared to RNs, who integrate HH into their daily routines within an established workplace culture (Labrague et al. 2018). Perceived barriers, such as a lack of time, inadequate resources and inconsistent role modelling, further influence adherence (Tan and Olivo 2015; Sopjani 2016). Additionally, the absence of structured feedback on infection prevention efforts has been highlighted as a challenge for HCWs, potentially impacting their ability to sustain proper HH practices (Dehghan Manshadi et al. 2022).

To better understand these differences, the WHO developed the ‘Perceptions Survey for Health‐Care Workers’, a validated tool for assessing HH attitudes (World Health Organization 2009; WHO 2009a). Studies utilising this survey have identified varied perceptions across different HCW groups, the impact of organisational support, and correlations between positive attitudes and higher HH adherence (Dehghan Manshadi et al. 2022; Oh 2019; Qasmi et al. 2018). However, no research directly comparing nursing students in different semesters and RNs has been found, despite their different professional stages and exposure to HH protocols.

Given this gap in knowledge, this study aims to compare HH perceptions and attitudes between nursing students and RNs, using the WHO Perceptions Survey for Health‐Care Workers. By examining these differences, we seek to provide insights into how HH training and workplace culture influence adherence at different stages of professional development.

## 3 The Study

### 3.1 Aim

The aim of this study was to describe and compare attitudes toward HH and the perceived effectiveness of prevention methods among nursing students and RNs at a Swedish university and its affiliated university hospital.

## 4 Methods

### 4.1 Design

A descriptive cross‐sectional total survey design was used to compare attitudes toward HH and perceived prevention effectiveness among nursing students and RNs. The WHO's ‘Perception Survey for Health‐Care Workers’ (WHO 2009a) served as the primary data collection instrument.

### 4.2 Study Setting and Sampling

This study was conducted at a Swedish university and an affiliated university hospital. Three distinct participant groups were targeted:

First‐semester nursing students (T1): In their first semester of a three‐year bachelor's nursing programme, they have received foundational infection prevention and control (IPC) education, including transmission routes, HH and sterilisation techniques. They also have had clinical placement experience, giving them exposure to HH practices in healthcare settings.

Final‐semester nursing students (T6): In their sixth and last semester. By this stage, they had advanced IPC knowledge, focusing on HAIs and quality control. All T6 met the qualification requirements to work as assistant nurses starting from the third semester of their training.

RNs: Clinically active in 12 wards (geriatrics, medicine, surgery) of the university hospital, which had an established IPC unit adhering to the WHO's Multimodal Hand Hygiene Improvement Strategy (WHO 2009b). The IPC unit provided the infrastructure for HH improvement, including training tools, evaluation and feedback mechanisms, reminders and ongoing work on patient safety issues. RNs had access to an interactive education programme on HH and dress code through the education intranet software, which they were expected to complete annually.

In total, 487 individuals were invited, including all T1 and T6 students and all RNs in the relevant departments. Response rates were 56% for T1 (*n* = 71), 57% for T6 (*n* = 46) and 30% for RNs (*n* = 84), with a total of 201 participants (41% overall response).

### 4.3 Instrument With Validity and Reliability/Data Source

Data were collected using the WHO's ‘Perception Survey for Health‐Care Workers’ (WHO 2009a), which underwent a forward‐backward translation into Swedish and was reviewed by an expert panel (World Health Organization, 2016). Some items were contextually adapted to fit the respondent group, such as replacing ‘your ward’ with ‘a hospital ward’ or adjusting the wording to reflect either student or professional clinical environments (e.g., use of ‘teacher’ vs. ‘supervisor’). These adaptations were made to keep the original meaning of the questions while making them easier to understand and more relevant for each group.

The survey included 24 questions derived from parts one and two of the original WHO perception survey, focusing on perceptions of HH, patient safety, HAIs and the effectiveness of measures aimed at improving HH.

Adjective descriptors were added after data collection, placed between the original endpoints of the Likert scales (Streiner et al. 2015). Subsequently, the survey results were organised into five distinct domains, each addressing specific aspects related to HH and healthcare perceptions:

1. HH and Patient Safety: Participants' perceptions of HH, assessed with three questions on a 4‐point Likert scale.
2. Effectiveness of HH Improvement Actions: Descriptions of the perceived effectiveness of various HH improvement measures, assessed with eight questions on a 7‐point Likert scale.
3. HH Practices and Perceptions in Healthcare Settings: The perceived importance of and effort related to HH practices within healthcare settings, assessed with four questions on a 7‐point Likert scale.
4. Perceived Support and Awareness in HH Improvement: Perceptions of support and awareness concerning HH improvement efforts, assessed with six questions on a 7‐point Likert scale.
5. Self‐reported HH Behaviour and Risk Perception: Evaluation of self‐reported HH behaviours and perception of infection risks, assessed with three numeric response questions ranging from 0 to 100%.

For detailed descriptions of each domain, Likert scale details and descriptors, see Data S1.

The questionnaire was pilot tested (*n* = 20, not included in the dataset), resulting in minor changes in phrasing.

### 4.4 Data Collection

Data were collected between December 2020 and January 2021, during the peak of the COVID‐19 pandemic, through a web‐based survey. Participants were accessed via institutional email lists obtained from the university and hospital administration. A week before the survey became available, an information letter explaining the study's purpose was sent to all eligible participants. Throughout the one‐month data collection period, two reminders were sent via e‐mail, 2 weeks apart. Nursing students received information through their student e‐mail accounts, while RNs received information through secretaries and department heads. T1 and T6 were also informed during an online lecture. Necessary permissions were obtained from the university and hospital authorities.

### 4.5 Data Analysis

Descriptive statistics were used to present the data, with values given as numbers, percentages, means (m) with standard deviations (SD) and medians (md) with interquartile ranges (IQR). The Kruskal–Wallis test was applied for all group comparisons to assess significant differences, given the ordinal nature of the data. When the Kruskal–Wallis test indicated a significant difference, post hoc Dunn's test was conducted to identify specific group differences, with *p*‐values adjusted for multiple comparisons using the Bonferroni correction. A *p*‐value of < 0.05 was considered statistically significant. Due to data skewness, non‐parametric statistical analyses were used.

Data were analysed using the Statistical Package for the Social Sciences version 28 (SPSS Inc., Chicago, IL, USA).

### 4.6 Ethical Considerations

This study adhered to the Declaration of Helsinki (World Medical Association 2013) and Swedish law (Riksdagsförvaltningen, n.d.). A specific approval from a research ethics committee was not required as there was no processing of sensitive personal data, and the study did not pose any physical or mental harm to participants. Necessary permissions were obtained from relevant authorities. The web‐based questionnaire included information about the study's purpose, voluntary nature and confidentiality. Furthermore, the participants were informed that by submitting the completed questionnaire, they consented to participate in the study. Participants received this information at the start of the questionnaire, before providing consent, all responses were anonymised and treated confidentially. This process ensured informed consent and the protection of participants' privacy.

## 5 Results

### 5.1 Characteristics of the Sample

Most of the participants were female (*n* = 169, 84.1%) and aged between 19 and 64 years, with a md age of 27 (23.00–34.75) (Table 1). Approximately half of the nursing students (*n* = 63, 55.3%) had prior experience working as an assistant nurse. The md duration of working as an RN was 9 (2.50–16.00) years.

**TABLE 1:** Demographic data of the study population. First semester students (T1), last semester students (T6) and registered nurses (RN).

Variable	T1 (*n* = 71)	T6 (*n* = 46)	RN (*n* = 84)
Age^a^	22 (20.00–25.25)	26 (23.00–29.25)	34 (28.00–46.75)
Female, *n* (%)	56 (78.9)	40 (87)	73 (86.9)
Male, *n* (%)	15 (21.1)	6 (13)	11 (13.1)
Experience of work as an assistant nurse, *n* (%)	29 (40.8)	34 (73.9)	
Working experience as a nurse^a^			9 (2.50–16.00)

^a^ Years, median (IQR).

### 5.2 Attitudes Toward HH

#### 5.2.1 HH and Patient Safety

The perceived impact of HAIs on patient clinical outcomes was high across all groups, with a mean (m ± SD) of 2.94 ± 0.54 for T1, 3.20 ± 0.58 for T6 and 3.10 ± 0.64 for RNs (Table 2; mds also provided). The effectiveness of HH in preventing HAIs was considered very high by T1 (3.86 ± 0.35), T6 (3.91 ± 0.29) and RNs (3.71 ± 0.55), with T6 considering it more effective than RNs (*p* = 0.049). Moreover, among all patient safety issues within the ward, the importance of HH was perceived as a very high priority by all groups (T1: 3.77 ± 0.49, T6: 3.61 ± 0.54, RNs: 3.39 ± 0.81), although T1 perceived it as more important than RNs (*p* = 0.003). For further details, see Data S2.

**TABLE 2:** Summary of Kruskal–Wallis test and post hoc Dunn's test with Bonferroni corrections for group responses among nursing student's semester 1 (T1) and semester 6 (T6) and registered nurses (RN) with mean (standard deviation [SD]) and median (interquartile range [IQR]) values displayed with respective dispersion for each group. Footnotes explain the range of each question.

Question	T1		T6		RN		Kruskal–Wallis *p*‐value	Dunn's test *p*‐value
	Mean (SD)	Median (IQR)	Mean (SD)	Median (IQR)	Mean (SD)	Median (IQR)		
Hand hygiene and patient safety								
Impact of a healthcare‐associated infection on a patient's clinical outcome^a^	2.94 (±0.54)	3 (3.00–3.00)	3.20 (±0.58)	3 (3.00–4.00)	3.10 (±0.64)	3 (3.00–3.00)	0.043	T1 vs. T6: 0.059 T1 vs. RN: 0.173 T6 vs. RN: 1.000
Effectiveness of hand hygiene in preventing healthcare‐associated infection^a^	3.86 (±0.35)	4 (4.00–4.00)	3.91 (±0.29)	4 (4.00–4.00)	3.71 (±0.55)	4 (3.00–4.00)	0.036	T1 vs. T6: 1.000 T1 vs. RN: 0.203 T6 vs. RN: 0.049
Importance is hand hygiene at a ward among all patient safety issues^b^	3.77 (±0.49)	4 (4.00–4.00)	3.61 (±0.54)	4 (3.00–4.00)	3.39 (±0.81)	4 (3.00–4.00)	0.004	T1 vs. T6: 0.267 T1 vs. RN: 0.003 T6 vs. RN: 0.718
Effectiveness of actions to improve hand hygiene adherence								
The healthcare facility ensures that alcohol‐based handrub availability^c^	6.66 (±0.74)	7 (6.75–7.00)	6.52 (±0.91)	7 (6.00–7.00)	6.47 (±0.93)	7 (6.00–7.00)	0.448	T1 vs. T6: 1.000 T1 vs. RN: 0.617 T6 vs. RN: 1.000
Leaders and senior managers at your institution support and advocate for hand hygiene^c^	6.01 (±1.15)	6 (5.00–7.00)	5.54 (±1.28)	6 (5.00–7.00)	5.51 (±1.48)	6 (5.00–7.00)	0.058	T1 vs. T6: 0.141 T1 vs. RN: 0.107 T6 vs. RN: 1.000
Each healthcare worker receives education on hand hygiene across^c^	6.66 (±0.61)	7 (6.00–7.00)	6.43 (±0.96)	7 (6.00–7.00)	6.18 (±1.17)	7 (6.00–7.00)	0.028	T1 vs. T6: 1.000 T1 vs. RN: 0.024 T6 vs. RN: 0.461
Visible simple and clear instructions for hand hygiene for every healthcare worker^c^	6.17 (±1.10)	7 (6.00–7.00)	5.87 (±1.38)	6 (5.00–7.00)	5.54 (±1.55)	6 (5.00–7.00)	0.035	T1 vs. T6: 0.787 T1 vs. RN: 0.029 T6 vs. RN: 0.757
Healthcare workers regularly receive feedback on their hand hygiene performance^c^	6.26 (±0.92)	7 (6.00–7.00)	5.89 (±1.65)	7 (5.00–7.00)	5.80 (±1.49)	6 (5.00–7.00)	0.252	T1 vs. T6: 1.000 T1 vs. RN: 0.312 T6 vs. RN: 1.000
You always perform hand hygiene as recommended (being a good example)^c^	6.27 (±1.01)	7 (5.75–7.00)	6.15 (±1.15)	6.5 (6.00–7.00)	6.10 (±1.04)	6 (5.00–7.00)	0.422	T1 vs. T6: 1.000 T1 vs. RN: 0.570 T6 vs. RN: 1.000
Hand hygiene posters are displayed at point of care as reminders^c^	5.46 (±1.48)	6 (4.75–7.00)	4.74 (±1.71)	5 (3.00–6.00)	4.60 (±1.69)	5 (3.00–6.00)	0.004	T1 vs. T6: 0.065 T1 vs. RN: 0.004 T6 vs. RN: 1.000
Patients are invited to remind healthcare workers to perform hand hygiene^c^	4.06 (±2.03)	4 (2.25–6.00)	4.55 (±2.23)	5 (2.25–7.00)	4.61 (±1.95)	5 (3.00–6.75)	0.221	T1 vs. T6: 0.579 T1 vs. RN: 0.313 T6 vs. RN: 1.000
Hand hygiene practices and perceptions in healthcare settings								
Organisational importance on optimal hand hygiene^d^	6.13 (±1.02)	6 (6.00–7.00)	5.69 (±1.28)	6 (5.00–7.00)	5.33 (±1.56)	6 (4.00–7.00)	0.005	T1 vs. T6: 0.234 T1 vs. RN: 0.004 T6 vs. RN: 0.905
Importance of peers and educators' commitment to optimal hand hygiene^d^	6.07 (±1.06)	6 (5.00–7.00)	5.15 (±1.40)	5 (4.00–6.25)	5.17 (±1.20)	5 (4.00–6.00)	0.001	T1 vs. T6 < 0.001 T1 vs. RN < 0.001 T6 vs. RN: 1.000
Importance that patients place on optimal hand hygiene^d^	5.55 (±1.44)	6 (5.00–7.00)	5.04 (±1.87)	5 (3.75–7.00)	4.19 (±1.81)	4 (3.00–6.00)	0.001	T1 vs. T6: 0.518 T1 vs. RN: < 0.001 T6 vs. RN: 0.025
Effort required to perform good hand hygiene^e^	3.23 (±1.74)	3 (2.00–4.00)	2.65 (±1.52)	2 (2.00–3.25)	2.34 (±1.63)	2 (1.00–3.00)	0.001	T1 vs. T6: 0.279 T1 vs. RN: 0.001 T6 vs. RN: 0.368
Perceived support and awareness in hand hygiene improvement								
Alcohol‐based handrubs tolerated by hands^f^	5.35 (±1.61)	6 (4.00–7.00)	5.76 (±1.48)	6 (5.00–7.00)	6.04 (±1.38)	7 (6.00–7.00)	0.009	T1 vs. T6: 0.442 T1 vs. RN: 0.007 T6 vs. RN: 0.699
Results of hand hygiene observations helping to improve hand hygiene^g^	6.13 (±0.91)	6 (6.00–7.00)	5.96 (±1.04)	6 (5.00–7.00)	5.84 (±1.22)	6 (5.00–7.00)	0.437	T1 vs. T6: 1.000 T1 vs. RN: 0.620 T6 vs. RN: 1.000
Impact of being observed and paying more attention to hand hygiene^g^	5.80 (±1.43)	6 (5.00–7.00)	5.45 (±1.52)	6 (5.00–7.00)	5.01 (±1.47)	5 (4.00–6.00)	0.001	T1 vs. T6: 0.935 T1 vs. RN: 0.001 T6 vs. RN: 0.067
Importance of educational activities to improve one's hand hygiene practices^h^	6.20 (±1.09)	7 (6.00–7.00)	5.70 (±1.42)	6 (5.00–7.00)	5.29 (±1.26)	5 (4.00–6.00)	0.001	T1 vs. T6: 0.142 T1 vs. RN: < 0.001 T6 vs. RN: 0.74
Perceived support from the management in improving hand hygiene^g^	6.41 (±0.88)	7 (6.00–7.00)	5.52 (±1.35)	6 (4.00–7.00)	5.23 (±1.48)	5 (4.00–7.00)	0.001	T1 vs. T6: 0.001 T1 vs. RN: < 0.001 T6 vs. RN: 0.984
Awareness of one's role in preventing HAI by improving hand hygiene practices^g^	6.26 (±1.15)	7 (6–7)	6.17 (±1.25)	7 (6.00–7.00)	5.95 (±1.27)	6 (5.00–7.00)	0.119	T1 vs. T6: 1.000 T1 vs. RN: 0.146 T6 vs. RN: 0.542
Self‐reported hand hygiene behaviour and risk perception								
Perceived risk of patients developing a HAI^i^	28.03 (±20.21)	20 (10.75–30.00)	34.33 (±21.70)	30 (18.75–60.00)	40.81 (±23.79)	35 (20.00–60.00)	0.005	T1 vs. T6: 0.298 T1 vs. RN: 0.003 T6 vs. RN: 0.631
How often healthcare workers comply with hand hygiene protocols^i^	76.35 (±12.94)	80 (70.00–85.00)	74.87 (±14.14)	80 (67.50–85.00)	85.56 (±11.34)	90 (80.00–90.00)	< 0.001	T1 vs. T6: 1.000 T1 vs. RN: < 0.001 T6 vs. RN: < 0.001
Self‐reported adherence to hand hygiene in required situations^i^	91.73 (±9.34)	95 (90.00–100.00)	92.27 (±7.78)	95 (90.00–98.00)	92.89 (±6.40)	95 (90.00–99.00)	0.959	T1 vs. T6: 1.000 T1 vs. RN: 1.000 T6 vs. RN: 1.000

^a^ 1 = very low, 4 = very high.

^b^ 1 = low priority, 4 = very high priority.

^c^ 1 = not effective, 7 = very effective.

^d^ 1 = no importance, 7 = very high importance.

^e^ 1 = no effort, 7 = a big effort.

^f^ 1 = not at all, 7 = very well.

^g^ 1 = not at all, 7 = very much.

^h^ 1 = not at all, 7 = very important.

^i^ 0%–100%.

#### 5.2.2 Effectiveness of Actions to Improve HH Adherence

All groups perceived accessibility to alcohol‐based handrub as very effective in permanently improving HH at their institution, with mean scores of 6.66 ± 0.74 for T1, 6.52 ± 0.91 for T6 and 6.47 ± 0.93 for RNs (Table 2). Similarly, receiving education was perceived as highly effective by all groups (T1: 6.66 ± 0.61, T6: 6.43 ± 0.96, RNs: 6.18 ± 1.17), with T1 perceiving education as a more effective action to improve HH compared to RNs (*p* = 0.024). For further details, see Data S3.

Simple and clear instructions were perceived as very effective in improving HH adherence by T1 (6.17 ± 1.10), and quite effective by T6 (5.87 ± 1.38) and RNs (5.54 ± 1.55). T1 found simple and clear instructions to be more effective compared to RNs (*p* = 0.029).

Receiving regular feedback and setting ‘a good example’ by always practising HH as recommended were considered very effective actions by T1 (6.26 ± 0.92) and T6 (5.89 ± 1.65), and quite effective by RNs (5.80 ± 1.49). However, the differences were not statistically significant.

T1 (5.46 ± 1.48) perceived the presence of HH posters as reminders to be quite effective compared to T6 (4.74 ± 1.71) and RNs (4.60 ± 1.69), who found them to be effective. T1 considered posters and reminders to be more effective than RNs (*p* = 0.004).

Patient involvement in reminding HCWs to practise HH was considered a moderately effective action by T1 (4.06 ± 2.03), and an effective action by T6 (4.55 ± 2.23) and RNs (4.61 ± 1.95). However, the differences were not statistically significant.

#### 5.2.3 HH Practices and Perceptions in Healthcare Settings

Regarding organisational priority, all groups perceived that the nursing programme or hospital management places high importance on maintaining optimal HH, with mean scores as follows: T1 (6.13 ± 1.02), T6 (5.69 ± 1.28) and RNs (5.33 ± 1.56) (Table 2). T1 perceived this as significantly more important compared to RNs (*p* = 0.004). For further details, see Data S4.

T1 (6.07 ± 1.06) rated the importance of their peers' and educators' commitment to optimal HH performance as high, while T6 (5.15 ± 1.40) and RNs (5.17 ± 1.20) rated it as moderately important. T1's perception of this importance was greater than both T6 (*p* < 0.001) and RNs (*p* < 0.001).

When considering the importance patients place on optimal HH, survey results indicate that T1 (5.55 ± 1.44) perceived this as high, T6 (5.04 ± 1.87) rated it as moderate and RNs (4.19 ± 1.81) considered it somewhat important. RNs rated the importance that patients place on optimal HH lower than both T1 (*p* < 0.001) and T6 (*p* = 0.025).

The effort required to perform good HH was considered low by T1 (3.23 ± 1.74) and very low by T6 (2.65 ± 1.52) and RNs (2.34 ± 1.63). RNs perceived a significantly lower effort required compared to T1 (*p* = 0.001).

#### 5.2.4 Perceived Support and Awareness in HH Improvement

The use of alcohol‐based handrubs was well tolerated by T1 (5.35 ± 1.61), T6 (5.76 ± 1.48) and very well tolerated by RNs (6.04 ± 1.38) (Table 2). RNs rated the use of alcohol‐based handrubs as more tolerable than T1 (*p* = 0.007). For further details, see Data S5.

All groups acknowledged that knowing the results of HH observations is very helpful for them and their colleagues in improving HH practices.

When considering the impact of being observed and paying more attention to HH, both T1 (5.80 ± 1.43) and T6 (5.45 ± 1.52) perceived it as having a significant impact, while RNs (5.01 ± 1.47) considered the impact somewhat lower. T1 found the act of being observed to have a greater impact than RNs (*p* = 0.001).

The importance of participating in educational activities to improve one's HH practices was rated very high by T1 (6.20 ± 1.09), high by T6 (5.70 ± 1.42) and somewhat important by RNs (5.29 ± 1.26). T1 rated educational activities as significantly more important than RNs (*p* < 0.001).

When evaluating perceived support from management in improving HH, T1 (6.41 ± 0.88) reported very high support, whereas T6 (5.52 ± 1.35) reported high support, and RNs (5.23 ± 1.48) perceived somewhat high support. T1 experienced significantly more support from management compared to both T6 (*p* = 0.001) and RNs (*p* < 0.001).

Awareness of one's role in preventing HAIs through improved HH practices was perceived as having increased considerably for both T1 (6.26 ± 1.15) and T6 (6.17 ± 1.25) and to a lesser extent for RNs (5.95 ± 1.27) during their education, with no significant differences among the groups.

#### 5.2.5 Self‐Reported HH Behaviour and Risk Perception

The perceived risk of patients developing a HAI was estimated at 28.03% ± 20.21% by T1, 34.33% ± 21.67% by T6 and 40.81% ± 23.79% by RNs. The perceived risk by T1 was significantly lower compared to RNs (*p* = 0.003). For further details, see Data S6.

In evaluating how often HCWs comply with HH protocols, T1 reported an adherence rate of 76.35% ± 12.94% and T6 reported 74.87% ± 14.14%. RNs perceived adherence to be significantly higher, at 85.56% ± 11.34%, compared to both T1 (*p* < 0.001) and T6 (*p* < 0.001).

Regarding self‐reported adherence to HH in required situations, T1 reported an adherence rate of 91.73% ± 9.34%, T6 reported 92.27% ± 7.78% and RNs reported 92.89% ± 6.40%. No significant differences were found between the groups.

## 6 Discussion

This study uniquely compares HH perceptions between nursing students and RNs. Consequently, direct comparisons with previous studies are only partially applicable.

The main findings indicate that first‐semester nursing students perceive HH as more critical compared to RNs, aligning with literature on the influence of education and clinical exposure (World Health Organization and WHO Patient Safety 2009; ICN—International Council of Nurses 2023). Additionally, research underscores a positive correlation between favourable HH attitudes and adherence rates among nursing students, which is influenced by the workplace setting and culture (Labrague et al. 2018). These findings align with previous research, reported in a systematic review, on behavioural and attitudinal influences on HH (Afework and Tamene 2025) and reflect broader findings in the literature, in various HCW groups that emphasise the impact of clinical experience, education and organisational support (World Health Organization and WHO Patient Safety 2009; Pittet et al. 2000; Zimmerman et al. 2020; Barrett and Randle 2008; Labrague et al. 2018; Linnik et al. 2024; Tan and Olivo 2015; Sopjani 2016; Ajzen 1991). However, these studies did not compare nursing students and RNs. Nursing students often perceive other HCWs as key influencers for HH adherence, driven by a desire to conform to the norms of the clinical environment (Tan and Olivo 2015). Such a trend suggests that the emphasis on infection control within the curriculum significantly impacts nursing students' perspectives (Zimmerman et al. 2020; Barrett and Randle 2008). This may also explain the observed discrepancy in perceptions regarding the extent to which HCWs perform proper HH. Nursing students did not perceive the adherence of HCWs to be as high as the RNs rated their own adherence. This discrepancy may, in part, reflect social desirability bias, particularly among RNs, who may overestimate their own adherence due to professional norms or expectations (Contzen et al. 2015). Since no observational data were collected, the possibility that self‐reports do not fully align with actual behaviour should be considered when interpreting these findings.

Furthermore, the findings in this study underscore the acknowledged effectiveness of HH improvement actions. All groups recognised the importance of accessibility to HH resources and education, as outlined in the WHO's guidelines (World Health Organization 2009). These findings resonate with systematic reviews, which affirm the effectiveness of diverse behaviour change determinants, such as education and resource availability, in promoting HH adherence (Labrague et al. 2018; Luangasanatip et al. 2015; Huis et al. 2012). Recent evidence also shows that localised training and continuous education significantly enhance both the perceived effectiveness and long‐term sustainability of HH practices (Saito et al. 2023). This highlights the need for ongoing education and continuous provision of resources.

Regarding the role of feedback and role modelling, the high value placed on both by both nursing students and RNs in this study aligns with previous research emphasising the critical role of leadership in fostering a safety culture (World Health Organization and WHO Patient Safety 2009; Sax et al. 2007). Regular feedback, along with HH practices demonstrated by management and senior staff, is crucial in reinforcing these habits. Nevertheless, there are challenges, including issues related to self‐efficacy, organisational support (Tan and Olivo 2015; Sopjani 2016) and the availability of resources (Qasmi et al. 2018). These findings highlight the need for active managerial involvement in both providing resources and upholding HH standards, which is also desired by HCWs in our previous qualitative study (Blomgren et al. 2021).

The perceived greater managerial support among students compared to RNs may reflect the educational focus on HH (Labrague et al. 2018). Bridging this gap in clinical practice is crucial, as it could lead to enhanced adherence to HH protocols and overall improvement in patient safety outcomes.

The findings of this study further shed light on perceptions of patient involvement in reminding HCWs to perform HH. Both nursing students and RNs perceived this strategy as only moderately effective, despite increasing attention to patient engagement within healthcare. This may indicate that healthcare professionals underestimate the potential influence of patient reminders, or that such involvement is not yet fully embedded in clinical routines. Structural barriers, such as power dynamics, time constraints, or unclear expectations, may also limit patients' ability or willingness to speak up. These findings highlight a gap between the ideals of patient‐centred care and current perceptions in practice, suggesting a need for organisational support and cultural change to empower patients as active partners in infection prevention (Eng et al. 2021). This suggests an opportunity to incorporate patient‐centred approaches into HH adherence strategies, potentially serving as an additional reinforcement mechanism (Landers et al. 2012).

This study adds to the understanding of how perceptions and attitudes toward HH differ between nursing students and RNs. These contrasts may reflect differences in perceived behavioural control, attitudes and subjective norms experienced by students and RNs. The disparities in education, experience and workplace environments between these groups provide valuable insights into the influence of these factors on HH behaviour, resonating with the principles of the Theory of Planned Behaviour (Ajzen 1991). Importantly, our findings identify specific gaps in support and resources which, if addressed, could significantly enhance favourable perceptions and attitudes toward HH practices.

The findings in this study can be related to the Theory of Planned Behaviour (Ajzen 1991), which proposes that behaviour is shaped by attitudes, subjective norms and perceived behavioural control. First‐semester students' strong belief in the importance and effectiveness of HH reflects the TPB component of attitudes. Their emphasis on the influence of educators and peers aligns with subjective norms, shaped by early expectations during training. Differences in perceived management support and the effort required for HH relate to perceived behavioural control, influencing how feasible adherence seems in practice. Students reported stronger institutional support and a clearer sense of their role in infection prevention, potentially enhancing their perceived ability to perform proper HH. In contrast, RNs reported lower perceived support and placed less emphasis on educational initiatives, which may reduce their sense of control, especially when compounded by external barriers such as workload, time constraints and inconsistent feedback. These findings align with evidence from infection control research highlighting how social and organisational factors, including feedback, workload and leadership, influence HH behaviour among HCWs (Afework and Tamene 2025).

Additionally, it is important to consider the context in which the data were collected, during the peak of the COVID‐19 pandemic in Sweden (OECD 2021). The heightened attention to infection prevention at that time may have influenced participants' perceptions, particularly among nursing students navigating both clinical training and global uncertainty. This context could help explain the especially strong endorsements observed in the student group. A follow‐up study conducted in the post‐pandemic period could offer valuable insight into how these perceptions have evolved and whether early attitudes are sustained or diminished over time.

The finding that first‐semester students reported more positive attitudes toward HH than RNs highlights the potential impact of early education and training. This may reflect the strong emphasis on infection prevention in academic settings and the prominent role of teachers and clinical role models (Zimmerman et al. 2020). According to the Theory of Planned Behaviour (Ajzen 1991), such influences relate to subjective norms, where students are motivated by the expectations of educators, clinical supervisors and peers. In contrast, the more moderate ratings among RNs suggest that these attitudes may shift over time as clinical realities, such as time pressure, workplace norms and inconsistent feedback, begin to influence practice. The higher perceived managerial support among students may also reflect differences between academic and clinical environments, where support structures are more visible and consistent. These findings raise the possibility that the transition from education to clinical practice may influence both subjective norms and perceived behavioural control, thereby affecting HH adherence.

### 6.1 Strengths and Limitations of the Work

A key strength of our methodology is the inclusion of both novice and more advanced nursing students alongside fully qualified RNs, thus capturing a spectrum of experience (Creswell and Creswell 2018). Additionally, the use of the WHO ‘Perception Survey for Health‐Care Workers’ a validated and widely recognised instrument for assessing perceptions and attitudes of HH further enhances the study's credibility (WHO 2009b). However, the post hoc addition of descriptors may have influenced interpretation (Streiner et al. 2015). Nevertheless, the aim was to enhance the comprehensibility of the data. Although minor contextual adaptations were made to tailor the survey to students and RNs, the original meaning and structure of the WHO questions were preserved, which limits the risk of measurement bias (Polit and Beck 2021). As the study relied on self‐reported data, the possibility of social desirability bias cannot be entirely excluded, particularly among RNs who may feel professional pressure to report high adherence (Contzen et al. 2015). However, this risk is likely reduced by the anonymous nature of the responses and the absence of personal contact with the researcher, both factors known to promote more honest reporting in internet‐based self‐report surveys (Polit and Beck 2021; Vésteinsdóttir et al. 2019).

The predominantly female sample reflects typical nursing demographics, contributing to the study's representativeness (Kearns and Mahon 2021). Despite this, the low internal dropout rate (1%) suggests that the perception assessment tool was effective, and the pandemic offered a unique context for studying HH practices under increased demand.

However, some limitations should be acknowledged. The response rate among RNs (30%) may have introduced selection bias, as non‐responders might systematically differ from participants, potentially limiting the representativeness and generalisability of the findings (Creswell and Creswell 2018; Petrie and Sabin 2020). This concern is particularly relevant in the context of the COVID‐19 pandemic, where elevated stress and workload may have reduced survey participation among nurses (Kisely et al. 2020; Nashwan et al. 2021).

While descriptive statistics identified significant differences, they cannot establish causality or fully explain the complex factors influencing HH behaviour. Future research should employ qualitative methods to gain deeper insights into the evolution of HH attitudes across educational and experiential levels (Creswell and Creswell 2018). Such qualitative findings can be used to develop hypotheses for further testing and to study the multifactorial influences on HH behaviour.

### 6.2 Recommendations for Further Research

Future research could focus on tracking changes in HH perceptions and practices over time, from nursing education to clinical practice, and integrate qualitative methods to uncover deeper behavioural drivers. These insights are crucial for bridging educational and clinical practice gaps, improving adherence and reducing HAIs.

### 6.3 Implications for Policy and Practice

The results of this study underscore the importance of continuously nurturing and reinforcing positive HH attitudes, particularly as new graduates enter the workforce. Hospital management and unit leaders should prioritise visible support, regular feedback and easy access to resources, such as alcohol‐based handrub, to ensure that theoretical ideals persist in clinical settings. Structured HH feedback sessions, peer mentorship programmes and leadership‐led reminders can reinforce adherence and strengthen a safety culture. These interventions may help bridge the gap between educational emphasis and clinical realities, particularly for newly graduated nurses. Nursing programmes could also coordinate with healthcare facilities to align academic teaching with workplace culture, bridging the gap between classroom learning and daily practice.

## 7 Conclusion

Comparing HH perceptions among nursing students and RNs revealed that first‐semester students exhibit particularly strong endorsements for HH's importance and the effectiveness of improvement actions, more so than final‐semester students or RNs. These differences may stem from varying levels of clinical exposure, perceived feedback and workplace realities, and can also be understood in relation to attitudes, subjective norms and perceived behavioural control, as outlined in the Theory of Planned Behaviour (Ajzen 1991). The findings reinforce the need for strategies that maintain positive attitudes formed during training and highlight the crucial roles of education, managerial support and a supportive organisational culture in promoting consistent HH adherence to reduce HAIs. Based on these findings, interventions such as structured feedback, peer mentorship and leadership‐led role modelling should be considered to sustain adherence over time. In addition, nursing programmes could collaborate more closely with clinical settings to better align academic preparation with workplace expectations. Future research should explore how perceptions, attitudes and behaviours differ across groups at similar stages of education or clinical practice, particularly in the post‐pandemic context. In addition, qualitative studies could provide deeper insight into perceptions, contextual influences and behavioural drivers behind HH behaviour.

## Author Contributions

Conceptualization: P.‐O.B., K.H. and L.H. Methodology: P.‐O.B., K.H. and L.H. Validation: P.‐O.B., K.H. and L.H. Formal analysis: P.‐O.B. with supervision of K.H., L.H. and J.W. Investigation: P.‐O.B. with supervision of K.H. and L.H. Data curation: P.‐O.B. and J.W. with supervision of K.H. and L.H. Writing – original draft: P.‐O.B. with supervision of K.H. and L.H. Writing – review and editing: P.‐O.B. with supervision of K.H., L.H. and J.W. Visualisation: P.‐O.B. with supervision of K.H., L.H. and J.W. Supervision: K.H. and L.H. Project administration: K.H. Funding acquisition: K.H.

## Disclosure

Statistical information: There is a statistician on the author team Johan Westerbergh. The author affirm that the methods used in the data analyses are suitably applied to the study design and context, and that the statistical findings have been implemented and interpreted correctly. The authors agree to take responsibility for ensuring the choice of statistical approach is appropriate and is conducted and interpreted correctly.

## Conflicts of Interest

The authors declare no conflicts of interest.

## Supporting information


Data S1.

## Data Availability

Data are available in Supporting Information.
