# Supplementary material for: Attitudes and Perceptions Toward Hand Hygiene Among Nursing Students and Nurses: A Cross‐Sectional Comparative Survey
Source: J Adv Nurs. 2025 May 28;82(3):2245–56. doi: 10.1111/jan.17076 (PMC12907585; doi:10.1111/jan.17076)
Supplement: Supplementary file 1 — Data S1. [file JAN-82-2245-s001.docx]

| Supplementary Data I. Definition of Likert-scales with 1–4 and 1–7 descriptors, including original endpoints and additional descriptors in between. The four different domains are presented along with their number of questions and respective Likert-scales. | | | | | | | | | | | | | | | | | | | | | | | | | |
| --- | --- | --- | --- | --- | --- | --- | --- | --- | --- | --- | --- | --- | --- | --- | --- | --- | --- | --- | --- | --- | --- | --- | --- | --- | --- |
| 1. Hand Hygiene and Patient Safety, three questions. | | | | | | | | | | | | | | | | | | | | | | | | | |
| 1 | | | | | 2 | | | | | | | | 3 | | | | | | | | | 4 | | | |
| Very low | | | | | Low | | | | | | | | High | | | | | | | | | Very high | | | |
| Low priority | | | | | Moderate priority | | | | | | | | High priority | | | | | | | | | Very high priority | | | |
| 2. Effectiveness of Hand Hygiene Improvement Actions, eight questions. | | | | | | | | | | | | | | | | | | | | | | | | | |
| 1 | | 2 | | | | | 3 | | | | 4 | | | | | 5 | | | 6 | | | | 7 | | |
| Not effective | | Slightly effective | | | | | Somewhat effective | | | | Moderately effective | | | | | Effective | | | Quite effective | | | | Very effective | | |
| 3. Hand Hygiene Practices and Perceptions in Healthcare Settings, four questions. | | | | | | | | | | | | | | | | | | | | | | | | | |
| 1 | | | 2 | | | | | 3 | | 4 | | | | | 5 | | | 6 | | | | | 7 | | |
| No importance | | | Very low importance | | | | | Low importance | | Somewhat important | | | | | Moderately important | | | High importance | | | | | Very high importance | | |
| No effort | | | Very low effort | | | | | Low effort | | Some effort | | | | | Moderate effort | | | High effort | | | | | A big effort | | |
| 4. Perceived Support and Awareness in Hand Hygiene Improvement, six questions. | | | | | | | | | | | | | | | | | | | | | | | | | |
| 1 | 2 | | | | | 3 | | | | | | 4 | | | | | 5 | | | | 6 | | | 7 | |
| Not at all | Very poorly | | | | | Poorly | | | | | | Somewhat poorly | | | | | Somewhat well | | | | Well | | | Very well | |
| Not at all | Very little | | | | | Little | | | | | | Somewhat little | | | | | Somewhat much | | | | Much | | | Very much | |
| Not at all | Very unimportant | | | | | Unimportant | | | | | | Somewhat unimportant | | | | | Somewhat important | | | | Important | | | Very important | |
| Supplementary Data II. Perceptions of healthcare workers regarding hand hygiene and patient safety. Presented as frequencies and percentages n (%) of participants’ responses on a scale ranging from 1 to 4 among nursing students semester 1 (T1), nursing students semester 6 (T6) and registered nurses (RNs). Each group is presented with the number of total answers (N= X). | | | | | | | | | | | | | | | | | | | | | | | | |  |
| Impact of a healthcare-associated infection on a patient's clinical outcome | | | | | | | | | | | | | | | | | | | | | | | | |  |
|  | | | | 1 (very low) | | | | | 2 | | | | | 3 | | | | | | 4 (very high) | | | | |  |
| T1 (N= 69) | | | |  | | | | | 12 (17.4) | | | | | 49 (71) | | | | | | 8 (11.6) | | | | |  |
| T6 (N= 46) | | | |  | | | | | 4 (8.7) | | | | | 29 (63) | | | | | | 13 (28.3) | | | | |  |
| RN (N= 81) | | | | 2 (2.5) | | | | | 7 (8.6) | | | | | 53 (65.4) | | | | | | 19 (23.5) | | | | |  |
| The effectiveness of hand hygiene in preventing healthcare-associated infection | | | | | | | | | | | | | | | | | | | | | | | | |  |
|  | | | | 1 (very low) | | | | | 2 | | | | | 3 | | | | | | 4 (very high) | | | | |  |
| T1 (N= 70) | | | |  | | | | |  | | | | | 10 (14.3) | | | | | | 60 (85.7) | | | | |  |
| T6 (N= 46) | | | |  | | | | |  | | | | | 4 (8.7) | | | | | | 42 (91.3) | | | | |  |
| RN (N= 83) | | | | 1 (1.2) | | | | | 1 (1.2) | | | | | 19 (22.9) | | | | | | 62 (74.7) | | | | |  |
| Among all patient safety issues, how important is hand hygiene at your institution? | | | | | | | | | | | | | | | | | | | | | | | | |  |
|  | | | | 1 (low priority) | | | | | 2 | | | | | 3 | | | | | | 4 (very high priority) | | | | |  |
| T1 (N= 70) | | | |  | | | | | 2 (2.9) | | | | | 12 (17.1) | | | | | | 56 (80) | | | | |  |
| T6 (N= 46) | | | |  | | | | | 1 (2.2) | | | | | 16 (34.8) | | | | | | 29 (63) | | | | |  |
| RN (N= 84) | | | | 3 (3.6) | | | | | 8 (9.5) | | | | | 26 (31) | | | | | | 47 (56) | | | | |  |

| Supplementary Data III. Perceptions of the effectiveness of actions to improve hand hygiene adherence. Presented as frequencies and percentages n (%) of participants’ responses on a scale ranging from 1 to 7 among nursing students semester 1 (T1), nursing students semester 6 (T6) and registered nurses (RNs). Each group is presented with the number of total answers (N= X). | | | | | | | |
| --- | --- | --- | --- | --- | --- | --- | --- |
| Leaders and senior managers at your institution support and openly advocate for hand hygiene | | | | | | | |
|  | 1 (not effective) | 2 | 3 | 4 | 5 | 6 | 7 (very effective) |
| T1 (N= 68) | 1 (1.5) |  |  | 5 (7.4) | 14 (20.6) | 18 (26.5) | 30 (44.1) |
| T6 (N= 46) |  | 1 (2.2) | 3 (6.5) | 3 (6.5) | 15 (32.6) | 11 (23.9) | 13 (28.3) |
| RN (N= 84) | 1 (1.2) | 2 (2.4) | 6 (7.1) | 9 (10.7) | 23 (27.4) | 12 (14.3) | 31 (36.9) |
| The healthcare facility ensures that alcohol-based handrub is always available at every point of care | | | | | | | |
|  | 1 (not effective) | 2 | 3 | 4 | 5 | 6 | 7 (very effective) |
| T1 (N= 70) |  |  | 1 (1.4) | 1 (1.4) | 2 (2.9) | 13 (18.6) | 53 (75.7) |
| T6 (N= 46) |  |  | 1 (2.2) | 1 (2.2) | 4 (8.7) | 7 (15.2) | 33 (71.7) |
| RN (N= 83) |  |  | 2 (2.4) | 2 (2.4) | 7 (8.4) | 16 (19.3) | 56 (67.5) |
| Each healthcare worker receives education on hand hygiene | | | | | | | |
|  | 1 (not effective) | 2 | 3 | 4 | 5 | 6 | 7 (very effective) |
| T1 (N= 70) |  |  |  |  | 5 (7.1) | 14 (20) | 51 (72.9) |
| T6 (N= 46) |  |  |  | 4 (8.7) | 3 (6.5) | 8 (17.4) | 31 (67.4) |
| RN (N= 84) |  | 2 (2.4) | 1 (1.2) | 5 (6.0) | 10 (11.9) | 20 (23.8) | 46 (54.8) |
| Clear and simple instructions for hand hygiene are made visible for every healthcare worker | | | | | | | |
|  | 1 (not effective) | 2 | 3 | 4 | 5 | 6 | 7 (very effective) |
| T1 (N= 71) |  |  | 2 (2.9) | 5 (7.2) | 9 (13) | 16 (23.2) | 37 (53.6) |
| T6 (N= 46) | 1 (2.2) |  | 2 (4.3) | 4 (8.7) | 7 (15.2) | 12 (26.1) | 20 (43.5) |
| RN (N= 80) | 1 (1.3) | 3 (3.8) | 7 (8.8) | 7 (8.8) | 14 (17.5) | 19 (23.8) | 20 (36.3) |
| Healthcare workers regularly receive feedback on their performance of hand hygiene | | | | | | | |
|  | 1 (not effective) | 2 | 3 | 4 | 5 | 6 | 7 (very effective) |
| T1 (N= 68) |  |  |  | 4 (5.9) | 10 (14.7) | 18 (26.5) | 36 (52.9) |
| T6 (N= 44) | 2 (4.5) |  | 2 (4.5) | 5 (11.4) | 4 (9.1) | 6 (13.6) | 25 (56.8) |
| RN (N= 83) | 2 (2.4) | 1 (1.2) | 5 (6) | 7 (8.4) | 10 (12.0) | 22 (26.5) | 36 (43.4) |
| You always perform hand hygiene as recommended (being a good example for your colleagues) | | | | | | | |
|  | 1 (not effective) | 2 | 3 | 4 | 5 | 6 | 7 (very effective) |
| T1 (N= 70) |  |  | 1 (2.2) | 5 (10.9) | 2 (4.3) | 15 (32.6) | 41 (58.6) |
| T6 (N= 46) |  | 1 (2.2) |  | 5 (10.9) | 2 (4.3) | 15 (32.6) | 23 (50) |
| RN (N= 83) |  | 1 (1.2) | 1 (1.2) | 3 (3.6) | 16 (19.3) | 25 (30.1) | 37 (44.6) |
| Hand hygiene posters are displayed at points of care as reminders | | | | | | | |
|  | 1 (not effective) | 2 | 3 | 4 | 5 | 6 | 7 (very effective) |
| T1 (N= 70) |  | 4 (5.7) | 3 (4.3) | 10 (14.3) | 17 (24.3) | 12 (17.1) | 24 (34.3) |
| T6 (N= 46) | 1 (2.2) | 4 (8.7) | 7 (15.2) | 9 (19.6) | 7 (15.2) | 9 (19.6) | 9 (19.6) |
| RN (N= 84) | 3 (3.6) | 7 (8.3) | 12 (14.3) | 18 (21.4) | 20 (23.8) | 7 (8.3) | 17 (20.2) |
| Patients are invited to remind healthcare workers to perform hand hygiene | | | | | | | |
|  | 1 (not effective) | 2 | 3 | 4 | 5 | 6 | 7 (very effective) |
| T1 (N= 68) | 10 (14.7) | 7 (10.3) | 12 (17.6) | 10 (14.7) | 10 (14.7) | 7 (9.9) | 12 (17.6) |
| T6 (N= 44) | 7 (15.9) | 4 (9.1) | 3 (6.8) | 6 (13.6) | 4 (9.1) | 8 (18.2) | 12 (27.3) |
| RN (N= 84) | 6 (7.1) | 9 (10.7) | 11 (13.1) | 13 (15.5) | 13 (15.5) | 11 (13.1) | 21 (25.0) |

| Supplementary Data IV. Perception of hand hygiene practices in healthcare settings. Presented as frequencies and percentages n (%) of participants’ responses on a scale from 1 to 7 among nursing students semester 1 (T1), nursing students semester 6 (T6) and registered nurses (RN). Each group is presented with the numbers of answers (N= X). | | | | | | | |
| --- | --- | --- | --- | --- | --- | --- | --- |
| What importance does the head of your department/ nursing programme attach to the fact that you perform optimal hand hygiene? | | | | | | | |
|  | 1 (none) | 2 | 3 | 4 | 5 | 6 | 7 (very high) |
|  | n (%) | | | | | | |
| T1 (N= 70) |  | 1 (1.4) | 1 (1.4) | 1 (1.4) | 13 (18.6) | 23 (32.9) | 31 (44.3) |
| T6 (N= 45) |  | 1( 2.2) | 2 (4.4) | 3 (6.7) | 14 (31.1) | 9 (20.0) | 16 (35.6) |
| RN (N= 81) | 1 (1.2) | 4 (4.9) | 5 (6.2) | 14 (17.3) | 15 (18.5) | 17 (21.0) | 25 (30.9) |
| What importance do your colleagues/ course mates/ teachers attach to the fact that you perform optimal hand hygiene? | | | | | | | |
|  | 1 (none) | 2 | 3 | 4 | 5 | 6 | 7 (very high) |
| T1 (N= 69) |  | 1 (1.4) |  | 2 (2.9) | 20 (29.0) | 13 (18.8) | 33 (47.8) |
| T6 (N= 46) |  | 3 (6.5) |  | 12 (26.1) | 14 (30.4) | 6 (13.0) | 11 (23.9) |
| RN (N= 81) |  | 1 (1.2) | 5 (6.2) | 19 (23.5) | 22 (27.2) | 22 (27.2) | 12 (14.8) |
| What importance do patients attach to the fact that you perform optimal hand hygiene? | | | | | | | |
|  | 1 (none) | 2 | 3 | 4 | 5 | 6 | 7 (very high) |
| T1 (N= 69) |  | 2 (2.9) | 6 (8.7) | 7 (10.1) | 16 (23.2) | 13 (18.8) | 25 (36.2) |
| T6 (N= 46) | 1 (2.2) | 4 (8.7) | 6 (13.0) | 9 (19.6) | 4 (8.7) | 5 (10.9) | 17 (37.0) |
| RN (N= 84) | 5 (6.0) | 14 (16.7) | 11 (13.1) | 19 (22.6) | 11 (13.1) | 13 (15.5) | 11 (13.1) |
| How do you perceive the effort required of you to practise good hand hygiene when caring for patients? | | | | | | | |
|  | 1 (no effort) | 2 | 3 | 4 | 5 | 6 | 7 (a big effort) |
| T1 (N= 70) | 11 (15.7) | 19 (27.1) | 11 (15.7) | 14 (20.0) | 7 (10.0) | 3 (4.3) | 5 (7.1) |
| T6 (N= 46) | 10 (21.7) | 17 (37.0) | 8 (17.4) | 6 (13.0) | 1 (2.2) | 3 (6.5) | 1 (2.2) |
| RN (N= 82) | 32 (38.1) | 26 (31.7) | 8 (9.8) | 4 (4.9) | 6 (7.3) | 4 (4.9) | 2 (2.4) |

| Supplementary Data V. Perception of hand hygiene practices in healthcare settings. Presented as frequencies and percentages n (%) of participants’ responses on a scale ranging from 1 to 7 among nursing students semester 1 (T1), nursing students semester 6 (T6) and registered nurses (RN). Each group is presented with the number of answers (N= X). | | | | | | | |
| --- | --- | --- | --- | --- | --- | --- | --- |
| Is the use of alcohol-based handrubs well tolerated by your hands? | | | | | | | |
|  | 1 (not at all) | 2 | 3 | 4 | 5 | 6 | 7 (very well) |
|  | n (%) | | | | | | |
| T1 (N= 69) | 1 (1.4) | 3 (4.3) | 8 (11.6) | 7 (10.1) | 11 (15.9) | 18 (26.1) | 21 (30.4) |
| T6 (N= 45) |  | 2 (4.4) | 3 (6.7) | 4 (8.9) | 5 (11.1) | 12 (26.7) | 19 (42.2) |
| RN (N= 84) |  | 3 (3.6) | 5 (6.0) | 3 (3.6) | 9 (10.7) | 19 (22.6) | 45 (53.6) |
| Can the results of observations on how hand hygiene is performed help you and the healthcare staff in improving your hand hygiene practices? | | | | | | | |
|  | 1 (not at all) | 2 | 3 | 4 | 5 | 6 | 7 (very much) |
| T1 (N= 68) |  |  |  | 5 (7.4) | 9 (13.2) | 26 (38.2) | 28 (41.2) |
| T6 (N= 45) |  |  | 2 (4.4) | 1 (2.2) | 10 (22.2) | 16 (35.6) | 16 (35.6) |
| RN (N= 82) | 1 (1.2) |  | 2 (2.4) | 8 (9.8) | 17 (20.7) | 23 (28.0) | 31 (37.8) |
| Has being observed led to you paying more attention to your hand hygiene practices? | | | | | | | |
|  | 1 (not at all) | 2 | 3 | 4 | 5 | 6 | 7 (very much) |
| T1 (N= 66) | 2 (3.0) | 1 (1.5) | 1 (1.5) | 6 (9.1) | 10 (15.2) | 20 (30.3) | 26 (39.4) |
| T6 (N= 46) | 2 (4.3) | 1 (2.2) | 1 (2.2) | 4 (8.7) | 9 (19.6) | 16 (34.8) | 13 (28.3) |
| RN (N= 82) | 1 (1.2) | 5 (6.1) | 6 (7.3) | 15 (18.3) | 22 (26.8) | 19 (23.2) | 14 (17.1) |
| Were the educational activities you participated in important for improving your hand hygiene practices? | | | | | | | |
|  | 1 (not at all) | 2 | 3 | 4 | 5 | 6 | 7 (very important) |
| T1 (N= 69) |  | 1 (1.4) | 2 (2.9) | 2 (2.9) | 7 (10.1) | 22 (31.9) | 35 (50.7) |
| T6 (N= 44) |  | 3 (6.8) |  | 4 (9.1) | 10 (22.7) | 10 (22.7) | 17 (38.6) |
| RN (N= 80) | 1 (1.3) | 2 (2.5) |  | 18 (22.5) | 22 (27.5) | 23 (28.7) | 14 (17.5) |
| Do you believe that the nursing programme/ administrators in your institution are supportive of improving hand hygiene? | | | | | | | |
|  | 1 (not at all) | 2 | 3 | 4 | 5 | 6 | 7 (very much) |
| T1 (N= 69) |  |  | 2 (2.9) |  | 6 (8.7) | 21 (30.4) | 40 (58.0) |
| T6 (N= 46) |  | 1 (2.2) |  | 9 (19.6) | 8 (17.4) | 12 (26.1) | 14 (30.4) |
| RN (N= 82) | 1 (1.2) | 2 (2.4) | 9 (11.0) | 11 (13.4) | 22 (26.8) | 16 (19.5) | 21 (25.6) |
| Has your awareness of your role in preventing healthcare-associated infections by improving your hand hygiene practices increased during your education? | | | | | | | |
|  | 1 (not at all) | 2 | 3 | 4 | 5 | 6 | 7 (very much) |
| T1 (N= 68) |  | 2 (2.9) |  | 3 (4.4) | 9 (13.2) | 13 (19.1) | 41 (60.3) |
| T6 (N= 46) | 1 (2.2) |  |  | 4 (8.7) | 5 (10.9) | 10 (21.7) | 26 (56.5) |
| RN (N= 84) | 2 (2.4) |  | 1 (1.2) | 6 (7.1) | 15 (17.9) | 24 (28.6) | 36 (42.9) |

| Supplementary Data VI. Self-reported Hand Hygiene Behaviour and Risk Perception. Answers presented as median percentage %. | | | |
| --- | --- | --- | --- |
|  | T1 | T6 | RN |
| In your opinion, what is the average percentage of hospitalised patients who will develop a healthcare-associated infection? | 30 (N=47) | 30 (N=46) | 35 (N=77) |
| To what extent do you think that healthcare workers perform proper hand hygiene, either through hand disinfection or hand rubbing? | 76 (N=40) | 80 (N=46) | 90 (N=81) |
| On average, in what percentage of situations requiring hand hygiene do you actually perform hand hygiene, either by handrubbing or handwashing? | 92.5 (N=42) | 95 (N=45) | 95 (N=81) |
